# An update on the Pauwels classification

**DOI:** 10.1186/s13018-016-0498-3

**Published:** 2016-12-12

**Authors:** Min Shen, Chen Wang, Hui Chen, Yun-feng Rui, Song Zhao

**Affiliations:** 1 School of Medicine, Southeast University, No. 87 Ding Jia Qiao, Nanjing, Jiangsu 210009 China; 2 Department of Orthopaedics, Zhongda Hospital, School of Medicine, Southeast University, No. 87 Ding Jia Qiao, Nanjing, Jiangsu 210009 China; 3 Department of Sports Medicine, Shanghai Jiao Tong University Affiliated Sixth People’s Hospital, 600 Yishan Road, Shanghai, 200233 China

**Keywords:** Femoral neck fracture, Pauwels classification, Measurement of Pauwels angle, Post-operative complications, Therapeutic guideline

## Abstract

**Background:**

Femoral neck fractures typically occur as a result of high-energy mechanisms among non-geriatric patients. Complications, including femoral neck shortening, non-union, and avascular necrosis, are relatively common after the internal fixation of this fracture pattern. These complications have serious effects on young patients. The Pauwels classification, which is the first biomechanical classification for femoral neck fractures, is still frequently used to determine and prescribe the appropriate treatment for femoral neck fractures. However, we lack a unified standard for measuring the Pauwels angle, which may make the classification unreliable. Understanding the relationship between the Pauwels classification and the complications arising from the internal fixation of femoral neck fractures is necessary. Meanwhile, a Pauwels type III femoral neck fracture among young adults, which involves a high shear load at the fracture site, is difficult to treat successfully. In addition, the recognized internal fixation for this fracture pattern remains uncertain.

**Main body:**

This review aims to provide an update on the viewpoint on the Pauwels classification including the measurement of the Pauwels angle and to present evidence to prove the aforementioned relationship. Moreover, this article also discusses the optimal internal fixation for femoral neck fractures based on the Pauwels classification.

**Conclusion:**

A unified standard of measurement should be established for the Pauwels classification, which is still frequently used in the literature and in determining appropriate treatment for femoral neck fractures, to achieve a credible classification. In addition, more randomized, multicentric, and prospective trials should be conducted in the future to clearly understand the relationship between the Pauwels classification and complications arising from the internal fixation of femoral neck fractures and, consequently, to explore ideal fixations for a Pauwels type III femoral neck fracture.

## Background

Femoral neck fractures typically occur in young patients as a result of high-energy trauma with a common pattern of a Pauwels type III fracture [1, 2]. Complications, including femoral neck shortening (FNS), non-union, and avascular necrosis (AVN), are relatively common after the internal fixation of this fracture pattern [3–9]. These complications will result in poor functional outcome and a high risk for reoperation and lifelong morbidity. The Pauwels classification, which is the first biomechanical classification for femoral neck fractures, is still frequently used at present [10, 11]. However, a series of misinterpretations and the lack of a unified standard for measuring the Pauwels angle may make the Pauwels classification unreliable [12–18]. The relationship between the Pauwels classification and the complications arising from the internal fixation of femoral neck fractures among young patients, which can assist in developing treatment plans, remains unclear. In the non-elderly population, the recognized internal fixation for the Pauwels type III femoral neck fracture in which shearing stress is dominant continues to be a popular topic. Therefore, future studies should consider these unsolved problems.

## Main text

### Definition of the Pauwels classification

**Fig. 1:**
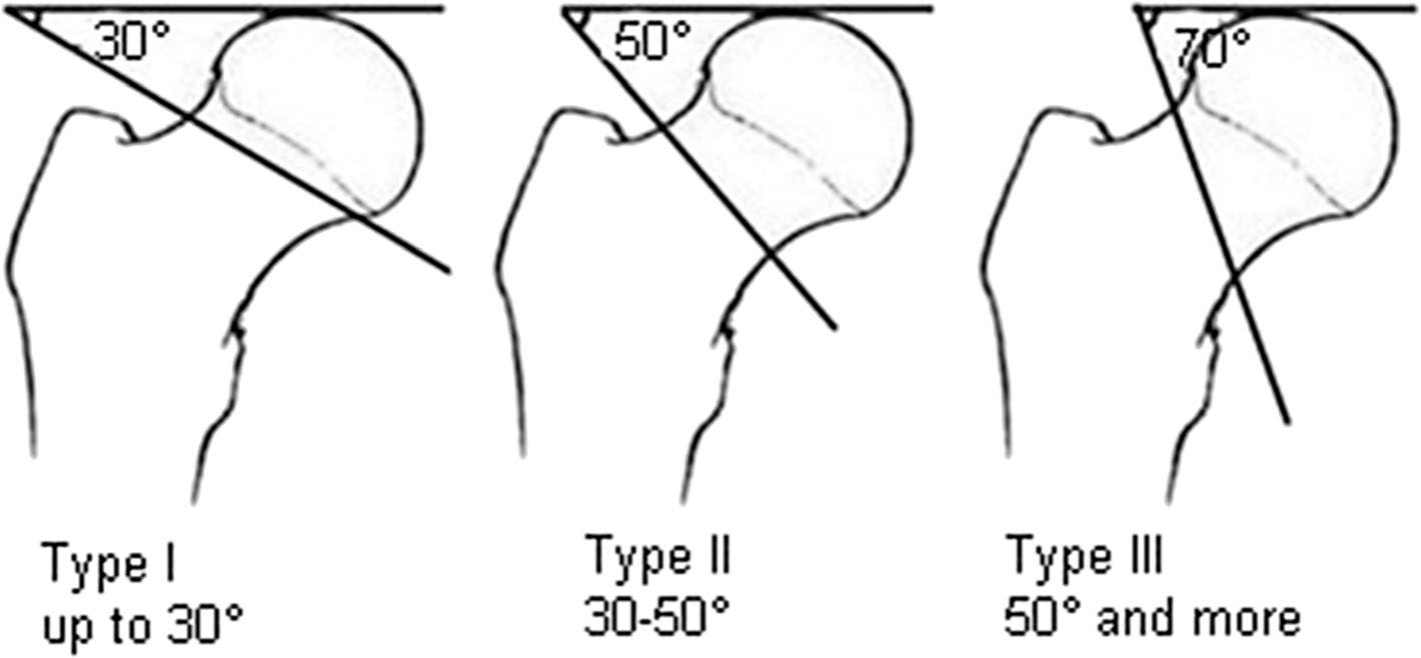
The Pauwels classification

The Pauwels classification, which was introduced in 1935, was the first biomechanical classification for femoral neck fractures [10]. This classification, which is still frequently used at present, calculates the angle between the fracture line of the distal fragment and the horizontal line to determine shearing stress and compressive force. The classification is described as follows (Fig. 1) [10, 11].

- Type I: up to 30°. Compressive forces are dominant.
- Type II: 30°–50°. Shearing force occurs and may have a negative effect on bone healing.
- Type III: 50° and more. Under these circumstances, shearing force is predominant and is associated with a significant amount of varus force which will more likely result in fracture displacement and varus collapse.

When the Pauwels classification was first published in 1935 in a German literature, a series of misinterpretations mainly related to the angle of inclination for different types of fracture sprung up [12–16]. The common mistake was considering the classification standard to be 30° and 70°. The main reason for such mistake was probably authors citing a secondary source that misinterpreted the original one. Moreover, these authors probably did not read the text in German where the numerical values appeared; meanwhile, Pauwels illustrated his classification without presenting any numerical value for different types of femoral neck fracture [11].

### Measurement of the Pauwels angle

**Fig. 2:**
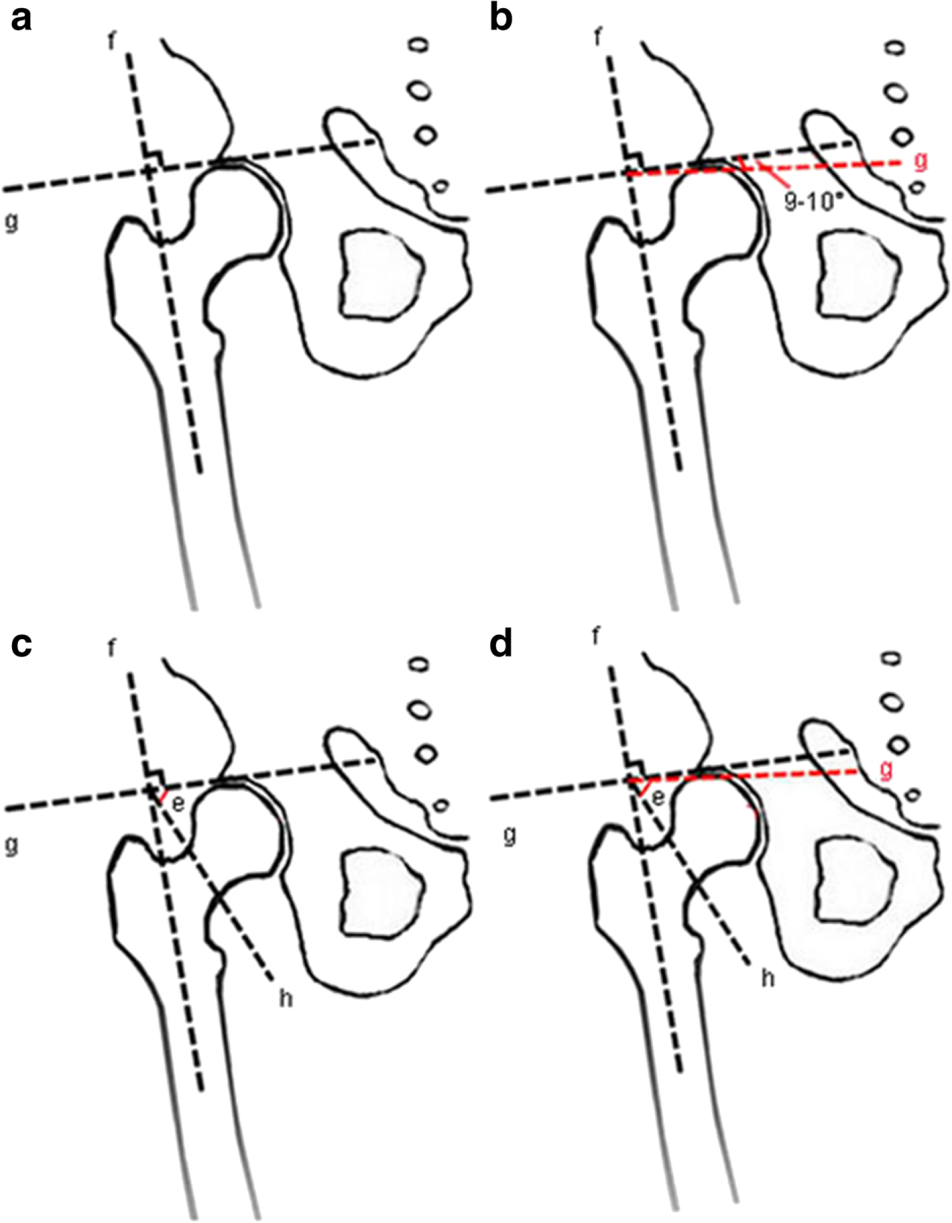
**a**–**d** Measurement of the Pauwels angle. Angle *e* represents the Pauwels angle; *f* is the line of the anatomic axis of the femoral shaft; *g* is the imaginary line which is equal to the horizontal line; and *h* is the line of fracture

The Pauwels classification for femoral neck fractures is still frequently used in the literature and is regarded as a therapeutic guideline in clinics. Recently, van Embden et al. investigated the reliability of this classification in preoperative planning [17]. The study asked five trauma surgeons and five surgical residents from two different medical centers who were familiar with the classification to independently classify 100 fractures based on the Pauwels classification using preoperative radiographs. They then calculated the Cohen kappa value to estimate inter-observer reliability. The result presented low inter-observer agreement with k0.31 (0.01), which indicated the unreliability of the classification. Gaspar et al. also identified the same problem and recommended the deprecation of the Pauwels classification [18]. The lack of a unified standard for measuring the Pauwels angle may make the Pauwels classification unreliable. The Pauwels angle, which consists of two lines (the horizontal line and the fracture line of the distal fragment) could be easily changed when the preoperative radiographs were taken because of the different positions of the leg, such as rotation and abduction [17, 19, 20]. The mutability of these two lines (the horizontal line and the fracture line of the distal fragment) in the radiographs can result in inaccuracy of the classification. Finally, the above studies showed the unreliability of the Pauwels classification. Therefore, the key to solving this problem is to set a unified standard for measuring the Pauwels angle which can confirm the aforementioned two lines. Recently, several studies have described a modification of the original Pauwels method [20, 21]. They used the anatomic axis of the femoral shaft as a guideline; then, they defined an imaginary line perpendicular to this guideline. The fracture line was drawn over the femoral neck to cross this line, and the modified Pauwels angle was defined as the angle between these two intersecting lines (Fig. 2a, c). In this modified method, the imaginary line is equal to the horizontal line. However, this new method appears to have defects. Under normal circumstances, an intersection angle of 6°–7° is observed when comparing the anatomic axis of the femoral shaft and the mechanical axis. Meanwhile, the mechanical axis and the gravity line intersect at 3° and the horizontal line is perpendicular to the gravity line. Thus, the imaginary line and the anatomic axis of the femoral shaft should intersect at 80°–81° and should not be perpendicular to each other (Fig. 2b, d). Therefore, more studies should focus on the measurement of the Pauwels angle, and a new unified standard should be established. Such standard can solve the unreliability problem and can improve the credibility of clinical outcome predictions.

### Complications

The complications arising from the internal fixation of femoral neck fractures, including FNS, non-union, and AVN, will result in a poor functional outcome and a high risk for reoperation and lifelong morbidity. The incidence of these complications remains relatively high despite the advancement in both knowledge and technologies [3–9]. Therefore, understanding the relationship between such complications and the Pauwels classification is vital to provide considerable assistance in selecting therapeutic schedules for femoral neck fractures. FNS after the internal fixation of a femoral neck fracture is common, and the incidence rate of FNS is high among both older adults and other age groups [3, 4]. Previous studies have demonstrated that FNS has a negative effect on the physical function of patients [3, 4]. At present, surgeons prefer to sacrifice the biomechanics of the hip to achieve biological healing of the fracture through compression of fracture fragments. This procedure will decrease the moment arm of the abductor muscles of the hip and significantly influences the functions and outcomes described in the arthroplasty literature [22, 23]. However, the relationship between the Pauwels classification and FNS is heterogeneous. The research of Stockton et al. on the predictors of FNS only showed a significant association between the initial fracture displacement and the fixation implant, with the exception of the Pauwels classification [24]. The primary limitation of current data is the retrospective non-randomized study design. Another important factor that should be considered in this study is the minimum necessary follow-up period of 6 weeks. By contrast, Zielinski et al. discovered that the degree of shortening increased as the age, weight, and the Pauwels classification of the fracture of the patient increased [25]. However, the effect of osteoporosis on FNS could not be determined because osteoporosis data were unavailable. In another study, age, Singh index, Pauwels classification, Garden alignment index, and body mass index were significantly associated with FNS greater than 5 mm [26]. Their retrospective nature and insufficient conclusions given the variable results are the major limitations of the preceding studies. Therefore, randomized, multicentric, and prospective trials should be conducted in the future to comprehend the aforementioned special relationship.

Non-union and AVN are not only the most significant sequelae of femoral neck fractures but are also the main reasons for reoperation. Researchers have never stopped studying the relationship between the Pauwels angle and non-union, which continues to be a source of debate. As Pauwels suggested in his original thesis, the more vertical the Pauwels angle is, the higher the incidence of non-union. However, the findings in the literature are heterogeneous. The study of Parker and Dynan, which was conducted among 335 patients, failed to find any correlation between the Pauwels angle and non-union in both displaced and undisplaced fractures [19]. However, the study found a significant association between the Pauwels angle and the Garden grade. This finding suggests that the more vertical the oriented line is, the more likely it will result in a displaced fracture. Similar results in which the Pauwels angle was not related to non-union risk were found in other studies [27–31]. By contrast, a recent study that used a modified method to predict the outcome of femoral neck fractures demonstrated that a highly modified Pauwels angle was a risk factor for non-union [20]. Meanwhile, Jo et al. described that the occurrences of non-union in the Pauwels type III fracture, subcapital-type fracture, and Garden stage III and IV fractures were higher than those of other types of femoral neck fracture with statistical significance [32]. Other previous literature found the same phenomenon with regard to the relationship between the Pauwels angle and non-union [14, 33–36].

In the AVN of the femoral head after the internal fixation of the femoral neck fracture, which is catastrophic for patients, Wang et al. indicated that higher modified Pauwels angles demonstrated significant differences with respect to AVN [20]. However, only a few studies have focused on the relationship between the Pauwels angle and AVN. Accordingly, future studies should focus on this major complication.

As can be seen from the above, according to the current evidence, we have not yet come to the exact relationship between the Pauwels classification and the complications arising from the internal fixation of femoral neck fractures. Therefore, we analyzed the reason behind the confusing result. One possible cause is that the Pauwels angle is mutable due to the different positions of the leg; it could result in the inaccuracy of the classification among different studies and the unreliability of the Pauwels angle. In other words, it means we lack a unified standard for measuring the Pauwels angle which can eliminate this kind of mutability. Therefore, a new unified method should be identified to standardize the measurement of the Pauwels angle, which will help in clearly understanding the relationship between the Pauwels angle and complications arising from femoral neck fractures.

### Therapeutic guideline

**Fig. 3:**
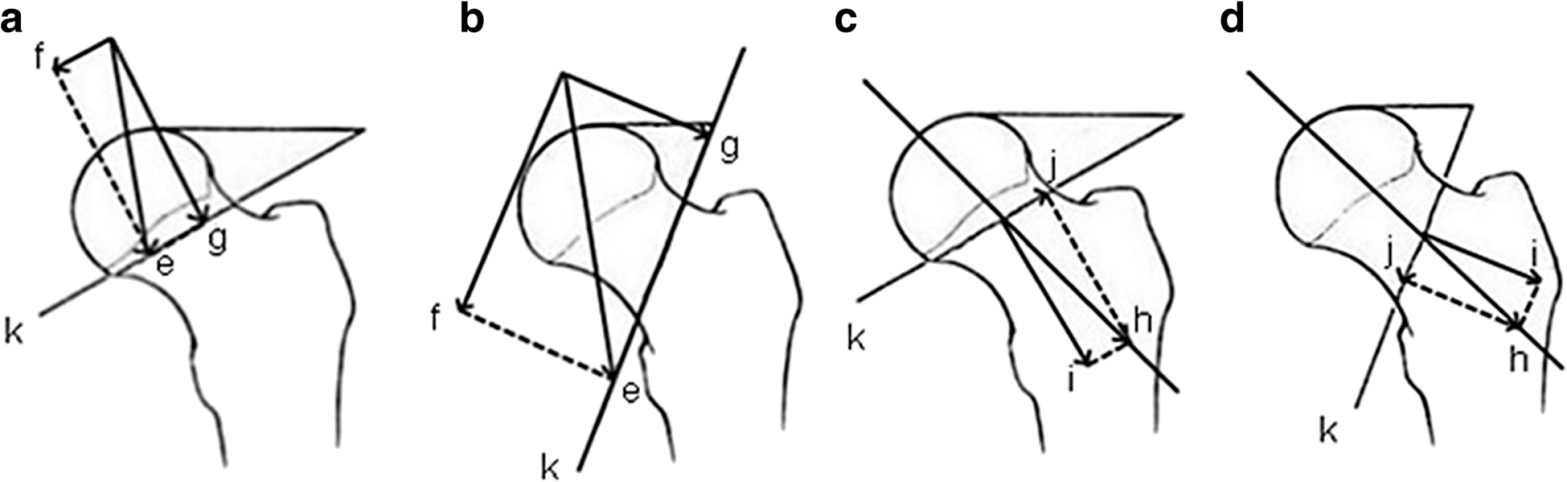
**a**–**d** Mechanical analysis. *e* denotes gravity, and *g* and *f* are the component forces of *e*; *h* is the force generated by the fixation, and *i* and *j* are the component forces of *h*; *k* is the line of fracture

Arthroplasty (hemiarthroplasty and total hip arthroplasty) is generally the best option for most older adults who sustained a displaced femoral neck fracture [16, 37]. By contrast, internal fixation is preferable among young adults who have a longer life expectancy and who wish to sustain their activity level [16, 37, 38]. The anatomical reduction and stable internal fixation of the femoral neck is the basis of managing femoral neck fractures among non-elderly patients to salvage the femoral head [39]. The options for internal fixation are varied and include cannulated screws, dynamic hip screw (DHS), cephalomedullary nails, and proximal femoral locking plates (PFLP). The Pauwels angle is still widely used in the literature and in preoperative planning. Several studies have suggested that Pauwels type I and II fractures, in which compressive forces are predominant, can be effectively managed with three parallel cannulated screws [16, 40] (Fig. 3a, c). However, the ideal fixation for the Pauwels type III fracture continues to be a popular topic.

Shearing forces, the component force of gravity, are dominant in the Pauwels type III fracture. Meanwhile, when internal fixations are fixed, they will generate not only compressive forces but also shearing forces (Fig. 3b, d). These forces will more likely result in fracture displacement and varus collapse. Therefore, the internal fixation for the Pauwels type III fracture should resist the vertical shearing force as much as possible. Numerous mechanical studies have investigated the ideal internal fixation for the Pauwels type III fracture (Table 1). However, no conclusion has yet been drawn. Selvan et al. compared six configurations of cannulated hip screws (including a triangle with superior, inferior, anterior, and posterior single screws and two or three vertical screws) in a biomechanical study, in which a model for the Pauwels type III fracture was built using synthetic bones [41]. The results clearly showed that the triangle configuration withstood higher levels of peak and ultimate loads, higher energy absorption, and less displacement compared with the other screw configurations. Hawks et al. assessed the biomechanics of a lag screw construct perpendicular to the fracture combined with two parallel cannulated screws [42]. The study suggested that using this construct to fix a vertically oriented femoral neck fracture provided substantial improvement in mechanical performance compared with the traditional inverted triangle construct. To imitate the method of Aminian et al. [1], Hawks compared their data and indicated that the stiffness of the trochanteric lag screw construct was similar to that of the DHS construct. Aminian et al. compared the biomedical stability of four different fixation techniques for vertical shear femoral neck fractures [1]. The study demonstrated the superior fixation stiffness of fixed-angle devices (e.g., PFLP, dynamic condylar screw) compared with that of cannulated screws for the fixation of Pauwels type III fractures in cadaveric specimens. Numerous studies have also demonstrated that fixed-angle constructs are superior to cannulated screws in biomechanics [2, 43, 44]. Meanwhile, research has not been limited to conventional fixations; many new constructs that exhibit biomechanical improvement compared with conventional fixations (e.g., cannulated screws, DHS) have also been developed [2, 44–47].

**Table 1:** Recommended internal fixations for the Pauwels type III femoral neck fracture

	Author	Year	Recommended internal fixation
Mechanics	Selvan	2004	The triangle configuration of cannulated hip screws
	Hawks	2013	A lag screw construct perpendicular to the fracture combined with two parallel cannulated screws
	Aminian	2007	Fixed-angle devices
	Nowotarski	2011	A novel femoral neck locking plate with two 5.7-mm locking head cancellous screws, one lag screw into the calcar, and two screws into the shaft
	Baitner	1999	Sliding hip screw
	Rupprecht	2011	The Intertan
	Saglam	2014	Minimal invasive sliding anti-rotator compressive hip screw
	Basso	2014	A lateral locking plate combined with three screws
	Samsami	2015	Dynamic hip screw with derotational screw
Clinic	Liporace	2008	Fixed-angle devices
	Chen	2011	A dynamic hip screw combined with an anti-rotation screw
	Virkus	2009	A horizontal lag screw combined with two parallel cannulated screws

Meanwhile, numerous clinical studies have also explored optimal fixation for Pauwels type III femoral neck fractures (Table 1). In a recent clinical report, Liporace et al. followed up on 62 patients with Pauwels type III femoral neck fractures. Among which, 37 cases were treated with cannulated screws, whereas 25 cases were treated with fixed-angle devices (e.g., DHS, cephalomedullary nail, or dynamic condylar screw) [48]. The results showed that the non-union rate was 19% for fractures treated with cannulated screws alone and 8% for those treated with a fixed-angle device and the difference between the two groups was insignificant. In another study, Chen et al. compared the curative effect of DHS combined with an anti-rotation screw and three cannulated screws in treating Pauwels type II or III femoral neck fractures, including the rates of non-union, implant failure, AVN, reoperation, overall success, union time, Harris hip score, and visual analog scale score [49]. The author suggested that the optimal fixation for the Pauwels type II or III femoral neck fracture among young adults was DHS combined with an anti-rotation screw. In another clinical study, Virkus et al. reviewed the results of 28 vertical femoral neck fractures treated with a horizontal construct combined with two parallel cannulated screws [42]. The results presented a healing rate of 86% and suggested the use of the trochanteric lag screw construct for vertical femoral neck fractures. From the preceding results, fixed-angle devices (e.g., DHS) apparently provided more solid fixation and better outcomes [1, 2, 43, 44, 48, 49], whereas multiple cancellous screws offered the advantages of less invasive surgery, such as a small incision, less blood loss, and a brief hospital stay [50]. Therefore, the ideal fixation for the Pauwels type III femoral neck fracture can be a combination of these two traditional internal fixations. More biomechanical and clinical studies should be conducted in the future before an ideal fixation can be confirmed.

## Conclusions

In conclusion, a unified standard of measurement should be established for the Pauwels classification, which is still frequently used in the literature and in determining appropriate treatment for femoral neck fracture, to achieve a credible classification. In addition, more randomized, multicentric, and prospective trials should be conducted in the future to clearly understand the relationship between the Pauwels classification and complications arising from the internal fixation of femoral neck fractures and, consequently, to explore ideal fixations for the Pauwels type III femoral neck fracture.

## Abbreviations

AVN: Avascular necrosis
DHS: Dynamic hip screw
PFLP: Proximal femoral locking plates
FNS: Femoral neck shortening
